# Patterns of Dietary Supplement Use among GBT2Q Men and Non-Binary Individuals in Canada

**DOI:** 10.3390/nu16162678

**Published:** 2024-08-13

**Authors:** Seyedeh Yasaman Ghazitabatabai, Manahil Zaid, Laura Forbes, Adam Davies, Ben Klassen, Nathan J. Lachowsky, Dalia El Khoury

**Affiliations:** 1 Department of Family Relations and Applied Nutrition, University of Guelph, Guelph, ON N1G 2W1, Canada; ghazitas@uoguelph.ca (S.Y.G.); mzaid@uoguelph.ca (M.Z.); forbesl@uoguelph.ca (L.F.); adam.davies@uoguelph.ca (A.D.); 2 Community Based Research Center (CBRC), Vancouver, BC V6Z 2H2, Canada; ben.klassen@cbrc.net; 3 School of Public Health and Social Policy, University of Victoria, Victoria, BC V8P 5C2, Canada; nlachowsky@uvic.ca

**Keywords:** GBT2Q, non-binary, supplements, use, reasons, sources of information, determinants

## Abstract

There is a lack of research regarding dietary supplement (DS) use among Gay, Bisexual, Transgender, Two-Spirit, Queer (GBT2Q) men and non-binary individuals, despite the higher prevalence of body image issues and societal pressure within this community. This study aimed to investigate patterns and predictors of DS use in this population, including types of DS used, sources of information, and reasons for DS use. A validated and anonymous online questionnaire was completed by 204 participants (52.5% men, 43.1% gay, mean age 29.34 + 6.77 years) across Canada, who were consuming DS at the time of the study. Analyses included descriptive statistics to characterize the sample based on gender, sexuality, age, ethnicity, and other demographic attributes, using Pearson’s chi-square tests and multi-way cross-tabulation analyses. Additionally, regression analyses, including binary and logistic regressions, were employed to identify predictors of DS use. Data analysis concluded that vitamins/minerals (92.2%), proteins (84.3%) and carbohydrates (75.5%) were the most consumed types of supplements. Identifying as certain genders and sexualities was significantly associated with supplement preferences, such that men reported higher use of amino acids (*p* = 0.033) and non-vitamins/mineral antioxidants compared to individuals identifying as other genders (*p* = 0.006). Moreover, bisexual participants consumed amino acids (*p* = 0.043) and carbohydrates (*p* = 0.026) more frequently when compared to non-bisexual participants. The most listed reason for DS use was to improve immunity (60.3%), with health care professionals being listed as the source of information by most participants (51.0%). Findings from this study can serve as a foundation for further research in this area and can guide the formulation and implementation of adequate policies targeting this underserved population.

## 1. Introduction

In Canada, dietary supplements (DS) are classified as natural health products and defined as naturally occurring substances used to restore or maintain health including vitamins, minerals, probiotics, herbal remedies, homeopathic medications, traditional medicines like traditional Chinese and Ayurvedic medicines, and other products like amino acids and essential fatty acids [1]. The use of DS is prevalent in Canada, with approximately 47.3% of the population reporting regular use of DS [2]. Yet, the DS sector is subject to limited regulations, resulting in inaccurate ingredient labeling, health claims with limited scientific evidence, and possible contamination with illegal substances [3,4]. Harmful effects have been observed regarding overuse or misuse of DS, with supplements such as creatine being linked to reliance, low mood, cardiovascular health risks, acne, gynecomastia, testicular atrophy, and testicular cancer [5]. Additionally, DS use can potentially lead to the use of other substances or drugs [6]. In fact, adverse physiological effects such as stroke, cancer and increased morbidity are associated with the overuse or misuse of the Appearance and Performance Enhancing Drugs and Supplements (APEDS) [7]. The APEDS include substances intended to improve physical appearance, general health or performance and include legal and over-the-counter DS (e.g., protein powders, vitamins, fat burners) as well as illicit Anabolic-Androgenic Steroids (AAS) [7]. Unfortunately, it is difficult to associate negative health effects with individual supplements and a substantial number of incidents must occur before a product is withdrawn from the market [8].

The intake of DS is influenced by a variety of factors such as gender, race, age, ethnicity [9], and education level [10]. High prevalence of DS use has been reported in certain populations such as athletes as a means to enhance performance [11]. Due to societal pressures to attain the ideal lean and muscular physique, men are more likely than women to use APEDS [12]. An understudied population for DS use is sexual and/or gender minority men and non-binary individuals. Sexual and/or gender minority men refer to men who do not identify as heterosexual and/or cisgender and include gay, bisexual, pansexual, transgender, Two-Spirit, queer (GBT2Q), non-binary individuals and other men who show attraction to or have sexual contact with people of the same or multiple genders [13,14,15]. According to the minority stress theory, sexual and gender minority individuals endure social stressors at disproportionately higher rates such as homophobia and/or transphobia, which can account for differences in health-related behaviors and outcomes [16]. Sexual minority men, specifically gay and bisexual men, are more likely to experience issues with their body image compared to heterosexual men [13,17,18,19]. Body image dissatisfaction reflects the degree to which individuals are disappointed with their appearance [20]. The sexual objectification theory suggests that sexual minority men are under higher pressure to achieve the ideal body (i.e., lean, and muscular) to attract sexual partners, causing continuous monitoring of one’s appearance that results in body image dissatisfaction [18]. GBT2Q men and non-binary individuals who are obese or overweight experience stigmatization, internalized homophobia, societal pressure, and decreased mental, sexual, and emotional health, resulting in implications for the well-being and health of this population [13]. Compared to heterosexual men, sexual minority men report a higher prevalence of purging behaviors (e.g., vomiting, using laxatives) and higher rates of fasting and using diet pills for weight loss purposes [21]. Body dissatisfaction results from the drive for increased masculinity, which is significantly associated with APEDS use [22,23].

Muscle dysmorphia is a psychological consequence of the desire for muscle growth [24]. Men with muscle dysmorphia partake in excessive quantities of strength training exercises, consume a large number of calories a day, engage in harmful eating habits such as binge eating and are more prone to consume AAS [15,24]. With the primary goal of increased muscle mass, the most consumed supplements by sexual minority men include whey protein, creatine supplements and AAS [25]. Exacerbating the effects of muscularity dissatisfaction among sexual minority males is social media [26]. Compared to heterosexual men, sexual minority males tend to be more susceptible to media images that promote masculinity due to higher rates of body image issues and lower levels of self-esteem [26,27]. Additionally, dating app use is positively correlated with muscle dissatisfaction and AAS use among GBT2Q men [26]. Sexual minority men who use dating apps demonstrate significantly increased chances of using protein powders, muscle-building supplements, diet pills, and laxatives [28]. Interestingly, a larger amount of recent sexual partners is positively associated with the use of AAS, synthetic performance-enhancing substances, protein, and creatine supplements as well as a greater likelihood of driven exercise [16]. Thus, sexual minority men are more likely to commit potentially unhealthy weight control behaviors to achieve their desired body image such as developing eating disorders and the misuse of DS [19,20].

Given the lack of studies exploring prevalence and determinants of dietary supplement use in this at-risk population, these findings highlight the importance of investigating DS use within the context of the GBT2Q and non-binary communities. It is crucial to identify underlying psychosocial and ecological stressors that influence their decision to use DS, as one way to minimize the harmful effects of inappropriate DS use [22]. The overall purpose of this research was to investigate DS practices among GBT2Q men and non-binary individuals in Canada, which consisted of exploring the types of supplements consumed, the frequency of using the supplements, the reasons for which this population is using DS, their sources of information regarding their DS and the potential predictors of DS use within this population.

## 2. Materials and Methods

### 2.1. Study Design

This project consisted of two phases. Phase one included gathering qualitative data from 15 GBT2Q men to understand their use and experiences with DS, their unique needs regarding health services, and the barriers related to their health and wellness. Semi-structured interviews were conducted and collected data were utilized to guide the quantitative phase of the research project. The second phase, which is the one reported on in this paper, was guided by a community-based participatory approach and consisted of a cross-sectional survey in collaboration with the Community Based Research Centre (CBRC). The CBRC’s primary community-based research effort includes the Sex Now Survey, the largest and longest study of GBT2Q+ men’s health in Canada.

### 2.2. Participants

In collaboration with CBRC, which helped with the recruitment process, participants were recruited from the different provinces and territories in Canada. The inclusion criteria included those who identified as GBT2Q men or non-binary individuals, resided in Canada, could complete the survey in English or French, were above the age of 18 and had taken any DS in the past 6 months. Participant recruitment was conducted through emails and online posters posted on the CBRC website, social media platforms (i.e., Instagram, Facebook, LinkedIn, X) and newsletter and by recontacting participants from the Sex Now Survey. The survey was also shared among academic departments and professional personnel at the University of Guelph, and study collaborators were encouraged to advertise the study within their respective institutions. All participants provided electronic consent prior to completing the online questionnaire. Upon completing the questionnaire, participants were given the chance to enter a raffle draw to win 1 of 100 gift cards valued at $10.

A total sample size of 223 participants was targeted, aiming to reach a power of 0.80 with an alpha of 0.05 and an effect size of 0.07 considering the 8 potential predictors of DS use (gender, sexuality, age, type of DS, frequency of supplement use, reason for using DS, source of information, and participants’ education level).

This project was approved by the Research Ethics Board at the University of Guelph (REB#22-05-006).

### 2.3. Questionnaire Development and Design

The survey was based on a questionnaire used in previous studies exploring DS use among varsity athletes and university students [11,29,30] and on the Sex Now survey [31]. Content validation was completed by 11 qualified evaluators recruited through the CBRC listserv. Evaluation criteria for all sections of the survey included brevity and length, response options, clarity, repetition, the precision of the language used, whether the scale is balanced, bias, double-barreled questions, appropriateness of the questions and question format. The survey consisted of Likert scale-based responses, multiple response options and open-ended questions. Three survey sections were developed, including (1) Eligibility (2) Demographics and (3) DS use.

The eligibility section included questions about participants’ gender, sexuality, age, DS use and province of residence within Canada. Section 2 included multiple choice questions relating to participant demographics, and included participants’ backgrounds, program majors, parents’ or guardians’ program education, and medical conditions. Participants were provided the option to choose not to disclose or to specify additional items not listed. Section 3 focused on participants’ DS habits. A list of DS was developed based on an in-depth scan of Ontario’s supplement market and current literature. Prohibited substances by the World Anti-Doping Agency (WADA) including anabolic steroids and other illegal hormones and drugs were excluded from the survey. A total of 13 categories of DS were selected, including vitamins/minerals, proteins, amino acids, carbohydrates, stimulants/energy boosters, non-vitamins/mineral antioxidants, fatty acids, herbs and botanicals, fat burners, meal replacements/weight gainers, nitrates/nitric oxide/vasodilators, prebiotics/probiotics, and digestive enzymes. Common supplements within each category were listed and participants had the option to enter any missing supplements. For each category, participants were asked to indicate whether they had experience with the DS category, specify the DS used and select the frequency and length of use. For example, the protein supplement category included “whey protein”, “casein protein”, “soy protein”, “protein bars”, “powders/shakes”, “other(s)” and “I do not use any of the supplements”. Next, the highest frequency of corresponding supplement use was selected from a timeline of once per week or less, 2–3 times per week, 4–5 times per week, and 6 times per week or more. Lastly, the length of supplementation was specified by 1 month or less, 1–2 months, 3–5 months, 6 months or more. The section also utilized Likert scale responses to inquire about motivational factors contributing to DS use and sources of information regarding DS. Intentions and attitudes towards DS use were assessed using statements such as: “In the next 6 months, I am planning to consume or keep consuming a DS” and “DS help achieve/maintain a balanced diet”. Additionally, Participants were asked to report the use, frequency, length, and reasons for non-dietary supplements use including anabolic steroids, injectable peptides, amphetamines, pro-hormones (steroid analogues) and ephedrine through an open-ended question. The section concluded with two questions about the potential side effects of DS and non-dietary supplements and one question about participants’ interest in learning more about DS.

### 2.4. Statistical Analysis

Statistical analysis was completed using Statistical Package for the Social Science (SPSS), version 28.0.1.1 and statistical significance was determined by a *p*-value of ≤0.05. Descriptive and frequency statistics were utilized to characterize the sample based on demographic attributes including but not limited to gender, sexuality, age, and ethnicity. Pearson’s chi-square tests using multi-way cross-tabulation analysis of variables were conducted to determine whether relationships existed between the categorical variables and supplement use practices including types of supplements used, reasons for use and sources of information regarding DS. As many GBT2Q men and non-binary individuals identified with more than one gender or sexual orientation, Pearson’s chi-square was conducted across binary variables that involved two mutually exclusive groups: (1) those identifying with a specific gender/sexuality vs. (2) those who did not identify with the corresponding gender/sexuality. Age was divided into five groups and tested as a categorical variable: 18–29 years, 30–39 years, 40–49 years, 50–59 years, and 60 years and above.

Types of DS were compared between the different gender, sexuality, and age sub-groups. Sources of information and reasons for DS use were independently tested using multiway crosstabulation analysis against gender, sexuality, and age. Binary and ordinal logistic regression models were created to test if gender, sexuality, age, ethnicity, dietitian visit, and presence of primary care provider predicted supplement use, frequency, and length of most commonly used DS. All assumptions of the statistical analysis were evaluated and verified prior to finalizing the regression results. To avoid overfitting in the regression model and due to low sample size, multivariate logistic regression was not performed and only individual logistic regression models for all predictors were conducted. Additionally, independent variables were transformed into binary entities to avoid overfitting and to increase the generalizability of the results.

Lastly, frequency statistics in SPSS were utilized to analyze other demographic information collected in Section 1 and Section 2 of the survey including reading nutritional labels, potential reasons for this behavior, and non-dietary supplement use.

## 3. Results

### 3.1. Characteristics of Participants

**Figure 1:**
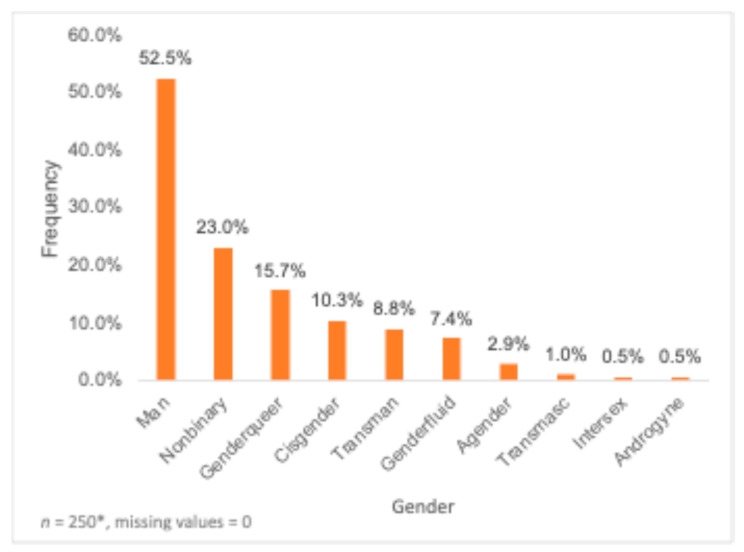
Distribution of sample by gender (*n* = 250). * Participants who chose more than one gender category were counted in each gender category that they indicated.

**Figure 2:**
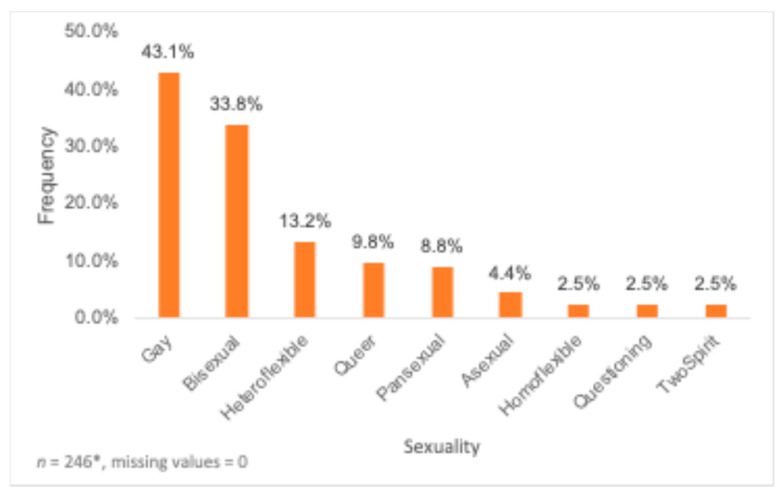
Distribution of sample by sexuality (*n* = 246). * Participants who chose more than one sexuality category were counted in each sexuality category that they indicated.

In total, 204 participants completed the online questionnaire. All responses were recorded, and no missing data were replaced. The mean age of the 204 participants who completed the survey was 29.34 ± 6.77 years, with most belonging to the 18–29 years (58.3%) and the 30–39 years (35.3%) age groups. Most participants identified as Gay (43.1%) and Men (52.5%) (Figure 1 and Figure 2). In total, 38 participants chose more than one gender category, and 28 participants chose more than one sexuality. However, participants who identified with more than one gender and/or sexuality group were counted in all the gender and sexuality groups that they have mentioned, independently. Most participants identified as White/European (65.7%) (Table 1) and lived in Ontario (32.4%), followed by Alberta (15.2%). Further, most participants did not report any medical conditions (69.1%), with the most common primary healthcare provider being a family doctor (42.6%) followed by a nurse practitioner (26.5%). Around 53.4% of participants had recently visited a dietitian, with 24.5% of participants reporting visiting a dietitian in the past 6 months while 21.1% of participants reporting not visiting a dietitian in the past 6 months (Table 1). A bachelor’s degree (45.6%), high school diploma (20.6%) and master’s or doctorate degree (13.7%) were the most completed levels of education (Table 1). Over a third of the participants (34.8%) reported the highest level of education of their parent or guardian being a bachelor’s degree, while 29.4% reported a high school diploma (Table 1).

### 3.2. Types of Used Dietary Supplements

Because of the eligibility criteria, all participants were supplement users. Table 2 summarizes the frequency of use of different categories of DS among the participants. Out of the 10 categories of DS, vitamins/minerals (92.2%), proteins (84.3%) and carbohydrates (77.5%) were the most commonly consumed. Further analysis of specific supplements within the vitamins/mineral category revealed that amongst 8 groups of vitamins/minerals supplements, the most consumed included vitamin C (45.6%), followed by vitamin B12 (36.3%), calcium (31.4%) and vitamin D (29.4%). Most participants indicated using at least one category of DS 2–3 times per week for 1–2 months. Those who were using herbs and botanicals were more likely to use DS less than once a week (31.8%) while participants who were using vitamins/minerals were more likely to be using DS more than 6 times a week (18.8%). The proportion of participants with less than a month of DS use was higher among those who were using fatty acids (21.9%). Lastly, the proportion of participants who were using DS for more than 6 months was higher among those who were using vitamins/minerals (31.7%).

Certain gender and sexual identities were significantly associated with the type of used DS, with those identifying as a man having a higher prevalence of amino acid (75.7%, *p* = 0.033), non-vitamin/mineral antioxidants (62.6%, *p* = 0.006), fatty acids (71.0%, *p* = 0.080), and herbs and botanicals (72.9%, *p* = 0.047) use when compared to individuals who did not identify as a man (Table 3). Additionally, identifying as cisgender was significantly associated with a higher prevalence of using carbohydrates (52.4%, *p* = 0.011) and a lower prevalence of using stimulants/energy boosters (23.8%, *p* < 0.001) and fat burners/weight loss supplements (38.1%, *p* = 0.022) compared to those who did not identify as cisgender (Table 3). Last, a significantly lower prevalence of stimulants/energy boosters (20.0%, *p* = 0.002) use was observed among participants who identified as genderfluid when compared to participants who did not identify as genderfluid (Table 3). Overall, a statistically significant difference was not present in the prevalence of use of vitamins/minerals and proteins among the different gender categories (Table 3).

In terms of sexuality, participants identifying as bisexual reported a significantly higher prevalence of using amino acids (78.3%, *p* = 0.043), carbohydrates (88.4%, *p* = 0.026), non-vitamins/mineral antioxidants (65.2%, *p* = 0.022), fatty acids (79.7%, *p* = 0.005), and herbs and botanicals (76.8%, *p* = 0.028) compared to participants who did not identify as bisexual (Table 4). Moreover, individuals who identified as pansexual reported a significantly lower prevalence of using amino acids (38.9%, *p* = 0.004), non-vitamins/mineral antioxidants (22.2%, *p* = 0.018), fatty acids (27.8%, *p* < 0.001), herbs and botanicals (44.4%, *p* = 0.036) and fat burners/weight loss supplements (33.3%, *p* = 0.012) compared to individuals who did not identify as pansexual (Table 4). In addition, individuals who identified their sexuality as homoflexible had a higher prevalence of consuming vitamins/minerals (60.0%, *p* = 0.005) with a lower prevalence of using protein supplements (40.0%, *p* = 0.006) and carbohydrate supplements (20.0%, *p* = 0.007) compared to individuals who did not indicate their sexuality to be homoflexible (Table 4). Last, those identifying as queer were significantly less likely to consume amino acids (30.0%, *p* < 0.001), stimulants/energy boosters (35.0%, *p* = 0.026), non-vitamins/mineral antioxidants (25.0%, *p* = 0.023), fatty acids (60.0%, *p* = 0.023), herbs and botanicals (40.0%, *p* = 0.008) and fat burners/weight loss supplements (40.0%, *p* = 0.044) compared to individuals who did not identify as queer (Table 4). Finally, a statistically significant association was not found between the sexualities of asexual, heteroflexible, questioning, and Two-Spirit and the use of any type of DS.

### 3.3. Reasons for Dietary Supplement Use

**Figure 3:**
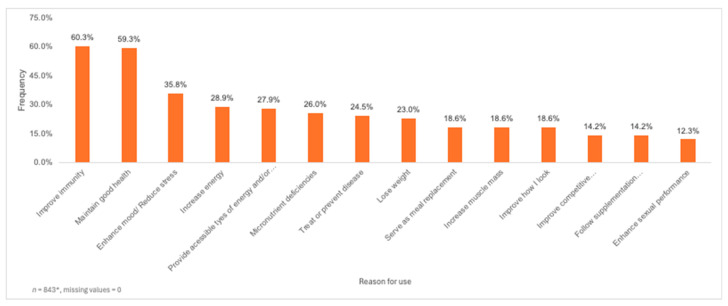
Reasons for using dietary supplements (*n* = 843). * Participants had the option to choose more than one reason for using dietary supplements.

Improving immunity (60.3%), maintaining good health (59.3%), enhancing mood and/or reducing stress (35.8%), increasing energy (28.9%), and providing accessible types of energy and/or micronutrients (27.9%), were the most frequently reported reasons for consuming DS (Figure 3). Comparing reasons for DS use by gender, non-binary participants (72.3%, *p* = 0.038) were significantly more likely to use DS for maintaining good health compared to participants identifying as other genders. Additionally, participants identifying as transmen were significantly more likely to consume DS to increase energy (50.0%, *p* = 0.039) compared to participants who did not identify as transmen. In terms of sexuality, participants who identified as queer (50.0%, *p* = 0.029) and questioning (80.0%, *p* = 0.011) had a significantly higher likelihood of using DS to increase energy levels when compared to participants identifying with other sexualities.

### 3.4. Source of Information

**Figure 4:**
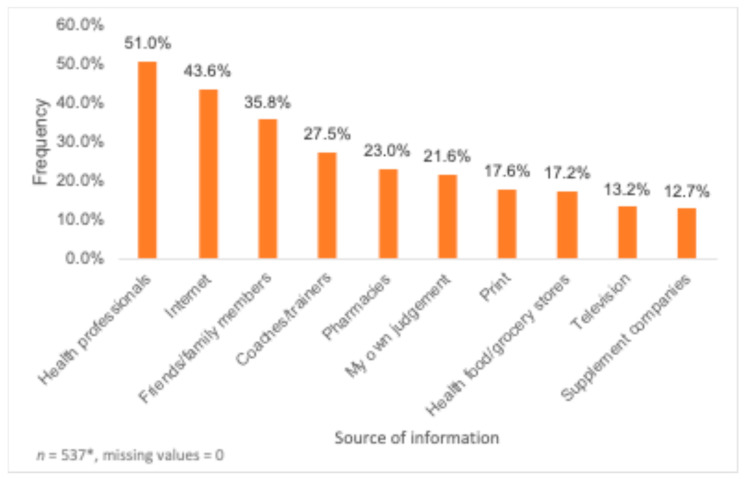
Sources of information regarding dietary supplements (*n* = 537). * Participants had the option to choose more than one source of information.

The most commonly listed sources of information regarding DS were health professionals (51.0%), the Internet (43.6), friends/family members (38.5%), coaches/trainers (27.5%), pharmacies (23.0%) and one’s own judgement (21.6%) (Figure 4). Gender, sexuality and age were found to have a significant association with sources of information. Genderqueer participants were significantly more likely to ask health professionals for information on DS use (68.8%, *p* = 0.029), while genderfluid participants were significantly less likely to do so (20.0%, *p* = 0.013) when compared to individuals from other gender categories. Identifying as a man was related to a significantly lower likelihood to refer to health food/grocery stores (12.1%, *p* = 0.046) when compared to individuals who did not identify as a man. Moreover, genderfluid participants were significantly less likely to ask their friends/family members (6.7%, *p* = 0.015) or to trust their own judgement (46.7%, *p* = 0.014) for information on DS compared to participants who did not identify as genderfluid. In addition, identifying as non-binary was associated with a significantly lower likelihood to refer to coaches/trainers (40.4%, *p* = 0.023) or to refer to information available in prints (4.3%, *p* = 0.006) for information on DS compared to individuals who did not identify as non-binary. Comparing sources of information by sexuality, queer individuals were found to have a significantly higher chance of trusting their own judgement (55.0%, *p* < 0.001) for information on DS compared to individuals who did not identify as queer. Finally, a statistically significant association for referring to the health food/grocery stores for information on DS was found for all age groups.

### 3.5. Predictors of Use of Dietary Supplements

Univariate, binary, and ordinal regressions were performed for the variables of gender, sexuality, age, ethnicity, healthcare provider and dietitian visit to identify factors that may predict or impact DS use, frequency, and length of use of DS among GBT2Q men and non-binary individuals. These variables were broken down into two levels with the most prevalent category being analyzed against the rest of the participants to increase the generalizability of the results and to avoid overfitting. Also, regression analysis was only performed for vitamins/minerals, proteins, and carbohydrate supplements given that these were the most commonly consumed DS by the participants.

A binary logistic regression model was estimated to investigate where an association existed between gender, sexuality, age, ethnicity, health care provider, dietitian visit, and participants’ vitamin, protein, and carbohydrate supplement use. The only significant models were for age and dietitian visit, with all other individual variables being unable to predict vitamin, protein, and carbohydrate supplement usage significantly (Table 5). Within this model, age was a significant predictor of protein supplement use (OR = 3.796, *p* = 0.038, 95%, CI: 1.165–12.469). Participants between the ages of 19–39 years old on average, had a higher likelihood of using protein supplements compared to participants in the 40–68 years old age group by a factor of 3.796. Overall, the model accounted for approximately 3.6% of the variance of protein supplement use, Ngelkerke R^2^ = 0.036. Dietitian visit was a significant predictor of vitamin, protein, and carbohydrate supplement use, independently (Table 5). On average, the models indicated that participants who visited a dietitian in the past 6 months had a lower likelihood of consuming vitamin (OR = 0.157, *p* < 0.001, CI: 0.052–0.469) and carbohydrate (OR = 0.122, *p* < 0.001, CI: 0.058–0.257) supplements. Overall, the models for dietitian visits in the past 6 months accounted for approximately 12.9% and 22.0% of the variance of vitamin and carbohydrate supplement use, respectively, Ngelkerke R^2^ = 0.129 vs. 0.220.

To estimate whether gender, sexuality, age, and ethnicity predicted the frequency of vitamin, protein and carbohydrate supplement use, an ordinal logistic regression analysis was run. Only ethnicity was found to be a significant predictor of the frequency of vitamin supplement use (OR = 0.535, *p* = 0.024, CI: 0.081–1.170), while dietitian visit was found to be a significant predictor of the frequency of carbohydrate supplement use. This model indicated that participants who visited a dietitian in the past 6 months had a higher frequency of carbohydrate use when compared to participants who did not visit a dietitian (OR = 0.160, *p* < 0.001, CI: 1.175–2.485). This model accounted for approximately 14.4% of the variance of frequency of carbohydrate supplement use, Ngelkerke R^2^ = 0.144.

An ordinal logistic regression analysis was conducted in order to estimate whether gender, sexuality, age, and ethnicity predicted the length of vitamin, protein and carbohydrate use. According to this model, ethnicity was found to be a significant predictor of length of vitamin supplement use with White/European individuals consuming vitamin supplements for longer durations of time compared to individuals with other ethnic backgrounds (OR = 0.455, *p* = 0.005, CI: 0.244–1.332) (Table 5). Overall, the model accounted for approximately 4.2% of the variance of length of vitamin supplement use, Ngelkerke R^2^ = 0.042. Moreover, ethnicity was also a significant predictor of length of protein supplement use with White/European participants consuming protein supplements for a longer period on average when compared to participants of other ethnic backgrounds (OR = 0.506, *p* = 0.013, CI: 0.143–1.219) (Table 5).

### 3.6. Nutritional Labels and Negative Side Effects

The majority of participants (62.7%) indicated always reading nutritional labels on DS, with 17.2% of participants reading nutritional labels sometimes and 16.7% of participants never reading the nutritional labels on DS. The most common reasons given by participants who indicated not reading or sometimes reading nutritional labels were trusting their own source of information (19.6%), not caring about food labels (8.8%) and lack of knowledge on how to read food labels (6.9%). In terms of negative side effects, most participants indicated never experiencing negative side effects related to DS usage. However, 12% of participants indicated experiencing a variety of gastrointestinal issues because of supplementation.

### 3.7. Use of Non-Dietary Supplements

Most participants (68.1%) indicated never having used non-dietary supplements while 21.6% of participants indicated using non-dietary supplements at the time of taking the survey. Non-dietary supplements were used less than once a week by 7.4% of total participants, while 12.7% of participants used these supplements 2–3 times per week. Amongst total participants, 5.9% indicated using non-dietary supplements for less than a month, 8.3% indicated 1–2 months, 7.8% stated 3–5 months and only 1.5% of participants stated using non-dietary supplements for more than 6 months. The most listed reasons for non-dietary supplement use were to enhance mood and/or reduce stress (12.3%), maintain good health (10.8%), treat, or prevent disease (7.8%), provide accessible types of energy and/or micronutrients (6.4%) and to lose weight (6.4%).

## 4. Discussion

This study is the first of its kind to explore the supplementation patterns and their determinants within the community of GBT2Q men and non-binary individuals in Canada. Overall, the most commonly consumed type of DS was found to be vitamin/mineral supplements, which is comparable to the general Canadian population [32]. The most used vitamins/minerals supplements were vitamin C and Vitamin B12. Considering that vitamin C and Vitamin B12 deficiencies are rare in Canada [33,34], the high prevalence of use of vitamin C and Vitamin B12 could potentially be attributed to the low risks associated with these supplements and/or misconceptions regarding their benefits. Next, protein supplements were the second most consumed category of DS, which agrees with results reported by Nagata et al. [16], where 42.5% of participants identifying as gay men indicated using protein supplements. In another study that explored the relationship between body image and supplement use among males, 49.9% of the participants were found to consume carbohydrate supplements (i.e., sports drinks), while 24.8% of participants reported consuming protein supplements (i.e., protein powders) to overcome body image concerns in the past two weeks [6]. Investigations on potential adverse outcomes of protein supplementation among this population are needed, considering that most North Americans receive enough protein from their diet without supplementation [35]. In light of the growing market of protein supplements and the widespread practice of consuming high-protein foods after workouts [32,36], further research should be conducted to determine the effectiveness of sex and gender-affirming health education interventions regarding DS usage among GBT2Q men and non-binary individuals. Considering that body image concerns may be driving protein supplementation, interventions focused on promoting body positivity could be helpful in reducing excessive dependence on protein supplements within GBT2Q men and non-binary individuals.

Our findings revealed that identifying as certain genders and sexualities was significantly associated with the types of DS used in this population. Individuals identifying as “man” were significantly more likely to use amino acid supplements. This is consistent with previous research, indicating a higher likelihood for men to use amino acid supplements for muscle growth or maintenance [37]. In addition, participants who identified as bisexual were significantly more likely to consume herbs and botanicals, which were most used to “reduce stress/enhance mood”. This could be attributed to the high level of societal stress experienced by sexual minority subgroups [16]. Most participants reported using DS for reasons other than muscle development or weight control, which demonstrates that GBT2Q men and non-binary individuals use DS for a wide range of reasons, not all of which are directly related to body image issues. This could be because participants in our study were more health cautious as evidenced by the higher prevalence of dietitian visits, which also suggested a higher socioeconomic status. In addition, these findings highlight the differences in the dietary practices within this population, which signify the importance of personalized approaches to better serve and educate GBT2Q men and non-binary individuals.

Most participants indicated using DS 2–3 times per week, which was lower than anticipated. The lower frequency of DS use within this sample can be attributed to the association between having higher education and subsequent exposure to well-developed nutrition educational programs on DS in schools and universities and the presence of a strong desire for “healthfulness” [30]. In addition, most participants indicated using DS for the duration of 1–2 months, which was lower than results from a similar study reporting that most participants used DS supplements for more than 6 months [30]. Further studies could investigate motivating factors for initiation and sustainment of DS use, considering that the association between frequency and length of DS use among GBT2Q men and non-binary individuals remains unclear. Many of the supplements may have been consumed in relation to physical activity, which can explain a non-daily basis of consumption.

Interestingly, health care providers were the most commonly listed sources of information for DS, which contradicts the findings from two studies [38,39] which found that the most popular sources of information among university students were friends and family, health food stores, and magazines and newspapers. However, certain studies involving university students do report health professionals as sources of information for the majority of the participants [30,40]. Healthcare professionals could therefore be leveraged to both educate and promote safe usage, specifically for vulnerable populations who are susceptible to DS use. Moreover, 44% of participants indicated using the Internet while 36% of participants indicated utilizing friends/family members as sources of information regarding DS, which raises concerns since the information distributed through the Internet and word of mouth is usually uncontrolled [30]. Furthermore, around 22% of participants reported using their own judgement as a source of information regarding DS, indicating that participants had a perceived understanding of DS but did not confirm its accuracy with reliable sources. As reported in another study [41], many individuals believe that DS are “natural” and do not have any negative side effects, explaining self-medication. These findings underscore the need for reliable sources of information that are easily accessible, with a focus on debunking misconceptions regarding DS usage. While participants in the present study who identified as genderqueer were significantly more likely to refer to health care professionals for information regarding DS, the relationship between sources of information on DS and gender and sexuality remains unclear.

The most common reasons for using DS supplements were “maintaining good health”, “increasing energy” and “improving immunity”, which is consistent with other studies conducted on Canadian young athletes and undergraduate students [39,42]. Another commonly reported reason for DS usage was to “enhance mood and/or reduce stress” (36%), which has not been investigated by previous studies to our knowledge. This is not surprising given the high prevalence of depression and anxiety among the GBT2Q men and non-binary population, due in part to stigma, discrimination, and minority stress [22]. This finding highlights the need for improved mental health support for this population. Moreover, in the current study, common reasons for taking DS were linked to the most commonly used DS such as vitamins/minerals, which were generally reported to “improve immunity”, “maintain good health”, and “provide accessible types of energy and/or micronutrients”. Also, protein supplements, commonly used in this population, were found to “increase or maintain muscle mass”, while using carbohydrate supplements was associated with the drive to “increase energy”. These findings showcase that the participants held perceived benefits associated with the type of DS being used, which can help healthcare providers better understand motivations for the use of various DS within GBT2Q men and non-binary individuals.

While identifying predictors of DS use among this community is of utmost importance to facilitate informed decision-making and policies to promote safe DS usage, data on such predictors specifically in regard to GBT2Q men and non-binary individuals are highly limited. In this current study, identifying as White/European was a significant predictor of DS usage. In addition, White/European participants reported a higher frequency and longer duration of DS use, which is consistent with results from other studies [9]. It is important to note that the definition of masculinity can shift in relation to an individual’s race or ethnicity [43], and individuals from different ethnic backgrounds likely have specific expectations regarding their bodies. Dietitian visit was another significant predictor of vitamin and protein supplement use with individuals who visited a dietitian in the past 6 months being less likely to consume these supplements. This is likely due to increased awareness regarding DS among participants who visit a dietitian and a “food first” strategy that is utilized by many nutrition experts. Further research is required to establish significant predictors of DS use among GBT2Q men and non-binary individuals. These findings suggest that both cultural norms associated with different ethnic and racial identities, as well as socioeconomic status and access to resources can influence DS usage. If similar predictors are found through future research, policies targeting GBT2Q men and non-binary individuals based on their cultural background and access to health information would be vital.

The present study is one of the first to explore dietary supplementation patterns and practices among the GBT2Q men and non-binary population in Canada. While there are various studies exploring DS use among males and females, GBT2Q men and non-binary individuals have been previously neglected in the literature. The strengths of this study include the use of a validated questionnaire that was tested on the target population, the extensive amount of information gathered regarding DS use, and the balanced representation of GB2TQ men and non-binary individuals from Canada. Contrarily, the limitations of this study include the self-reported nature of the data collected, which could be influenced by social desirability bias and memory errors during recall. To minimize social desirability bias, the anonymous nature of the questionnaire was emphasized. However, due to the anonymity of the survey, it was not possible to track all duplicate answers. To avoid duplicate responses, the survey was only allowed one submission per device and some of the methods mentioned in the study by Storozuk et al. [44] including checking the duration/time of survey completion and checking open text questions to detect duplicate or bot responses were utilized. Furthermore, since participation in the study was voluntary, the results may have been influenced by the fact that participants were generally more educated and health-conscious, as evidenced by their level of education and frequent visits to a registered dietitian. This might have affected the study’s outcomes. Additionally, the low presence of individuals from different gender, sexuality, and race categories greatly impacted the generalizability of our results regarding gender, sexuality, and race-based differences in DS use. Lastly, changing some of the variables to binary entities to avoid overfitting could have impacted the results of the logistic regressions.

## 5. Conclusions

This study is one of the first to provide insights regarding patterns and predictors of dietary supplement use among GBT2Q men and non-binary individuals in Canada, contributing to higher standards for equitable and robust research in this area. While many participants obtained information regarding dietary supplements from health professionals, a significant proportion of participants relied on unreliable sources including the Internet and friends and family. This finding highlights the importance of designing programs and policies that address the specific needs of the GBT2Q men and non-binary population. Future policies that target GBT2Q men and non-binary individuals based on their ethnic backgrounds and access to health professionals for information should be considered. Young adults continue to rely on DS irrespective of micronutrient status, while ignoring the lack of scientific rigor and risk of harmful effects associated with DS use [45]. These findings can bridge the gap in the literature to identify determinants and predictors of DS usage among GBT2Q men and non-binary individuals. Furthermore, health care professionals can utilize this information to design effective and inclusive educational materials on supplementation.

This study serves as a foundation for future research on GBT2Q men and non-binary individuals and their experiences with dietary supplements, and advocates for the development of educational programs related to safer use of dietary supplements.

## Figures and Tables

**Table 1:** Characteristics of participants (*n* = 204).

Characteristics		Percentage
Ethnicity	Arab (Saudi Arabian, Palestinian, Iraqi, etc.)	1.5%
	Black/African/Caribbean	5.9%
	East/Southeast Asian (e.g., Chinese, Japanese, Korean, Vietnamese, Cambodian, Filipino, etc.)	4.4%
	Indigenous (First Nations/Métis/Inuk)	8.4%
	Latin American, Latina, Latino, Latinx, Latine (Costa Rican, Guatemalan, Brazilian, Columbian, etc.)	1.5%
	Middle Eastern	1.0%
	South Asian (East Indian, Sri Lankan, etc.)	2.5%
	West Asian (Iranian, Afghani, etc.)	0.5%
	White/European	65.7%
	Mixed Ethnicity	7.8%
Dietitian visit in the last 6 months	Currently visits a dietitian	53.4%
	Has visited a dietitian in the past 6 months	24.5%
	Has not visited a dietitian in the past 6 months	21.1%
Highest level of education	Did not finish high school	2.0%
	Apprenticeship training/trades	5.4%
	Professional degree	11.3%
	Highschool diploma	20.6%
	Bachelor’s degree	45.6%
	Master’s or doctorate degree	13.7%
Parent’s/guardian’s highest level of education	Did not finish high school	3.9%
	Apprenticeship training/trades	7.4%
	Professional degree	13.7%
	Highschool diploma	29.4%
	Bachelor’s degree	34.8%
	Master’s or doctorate degree	9.3%

**Table 2:** Types of used dietary supplements, frequency of use, and length of use.

	Vitamins/Minerals	Proteins	Amino Acids	Carbohydrates	Stimulants/Energy Boosters	Non-Vitamin/Mineral Antioxidants	Fatty Acids	Herbs and Botanicals	Fat Burners/Weight Loss
Yes	92.2% (188) *	84.3% (172) *	69.1% (141) *	77.5% (158) *	62.3% (127) *	52.0% (107) *	65.2% (133) *	66.7% (136) *	64.7% (132) *
No	7.8% (16) *	15.7% (32) *	30.9% (36) *	22.6% (46) *	37.3% (76) *	46.1% (94) *	32.8% (67) *	33.3% (68) *	34.8% (71) *
>1 week	14.5% (27) *	21.0% (36) *	31.2% (44) *	17.3% (27) *	26.8% (34) *	27.6% (29) *	29.3% (39) *	31.8% (43) *	22.7% (30) *
2–3 times/week	41.4% (77) *	49.7% (85) *	52.5% (74) *	55.1% (86) *	46.5% (59) *	47.6% (50) *	47.3% (63) *	48.1% (65) *	48.5% (64) *
4–5 times/weeks	25.30 (47) *	25.4% (36) *	14.2% (20) *	21.1% (33) *	18.1% (23) *	19.0% (20) *	15.8% (21) *	15.5% (21) *	19.7% (26) *
<6 times/week	18.8% (35) *	8.20% (14) *	2.1% (3) *	6.4% (10) *	8.7% (11) *	5.7% (6) *	7.5% (10) *	4.4% (6) *	9.1% (12) *
>1 month	9.5% (18) *	12.8% (22) *	19.7% (28) *	13.4% (21) *	13.4% (17) *	15.2% (16) *	21.9% (29) *	14.2% (19) *	15.3% (20) *
1–2 months	33.3% (63) *	35.5% (61) *	45.1% (64) *	31.2% (49) *	37.8% (48) *	43.8% (46) *	31.1% (41) *	40.3% (54) *	41.2% (54) *
3–5 months	25.4% (48) *	23.2% (40) *	25.3% (36) *	27.4% (43) *	26.8% (34) *	32.4% (34) *	29.5% (39) *	25.2% (34) *	27.5% (36) *
<6 months	31.7% (60) *	28.5% (49) *	9.9% (14) *	28.0% (44) *	22.0% (28) *	8.6% (9) *	17.4% (23) *	20.1% (27) *	16.0% (21) *

* Numbers in the parenthesis reflect *n* = frequency.

**Table 3:** Percentage of participants using each type of dietary supplements based on gender.

	Non-Binary	Transman	Genderfluid	Man	Genderqueer	Cisgender
Vitamin	97.9% (0.118)	100.0% (0.211)	86.7% (0.357)	94.4% (0.316)	93.8% (0.795)	85.7% (0.199)
Proteins	83.0% (0.774)	83.3% (0.905)	86.7% (0.795)	86.9% (0.283)	90.6% (0.285)	81.0% (0.655)
Amino acids	63.8% (0.371)	50.0% (0.066)	86.7% (0.126)	75.7% (0.033) *	68.8% (0.961)	57.10% (0.210)
Carbohydrates	83.0% (0.082)	66.7% (0.465)	66.7% (0.534)	79.4% (0.485)	78.1% (0.091)	52.4% (0.011) *
Stimulants/energy boosters	68.1% (0.101)	61.1% (0.944)	20.0% (0.002) *	66.4% (0.287)	56.3% (0.658)	23.8% (<0.001) *
Non-Vitamin/Mineral Antioxidants	40.4% (0.167)	44.4% (0.632)	60.0% (0.762)	62.6% (0.006) *	50.0% (0.695)	38.1% (0.281)
Fatty acids	68.1% (0.878)	61.1% (0.720)	73.3% (0.715)	71.0% (0.080) *	62.5% (0.595)	52.4% (0.272)
Herbs and botanicals	57.4% (0.126)	55.6% (0.295)	73.3% (0.569)	72.9% (0.047) *	75.0% (0.276)	61.9% (0.625)
Fat burners/weight loss	61.7% (0.741)	55.6% (0.644)	73.3% (0.751)	71.0% (0.072)	71.9% (0.614)	38.1% (0.022) *

* Numbers in the parenthesis represent Chi-Square test *p*-values. *p*-values < 0.05 show statistically significant difference.

**Table 4:** Percentage of participants using each type of dietary supplements based on sexuality.

	Gay	Bisexual	Pansexual	Heteroflexible	Homoflexible	Queer
Vitamin	95.5% (0.181)	95.7% (0.240)	88.9% (0.522)	92.6% (0.991)	60.0% (0.005) *	90.9% (0.633)
Proteins	89.8% (0.062)	87.0% (0.458)	77.8% (0.425)	85.2% (0.894)	40.0% (0.006) *	75.0% (0.228)
Amino acids	70.5% (0.719)	78.3% (0.043) *	38.9% (0.004) *	77.8% (0.296)	100.0% (0.13)	30.0% (<0.001) *
Carbohydrates	75.0% (0.435)	88.4% (0.026) *	72.2% (0.795)	77.8% (0.926)	20.0% (0.007) *	60.0% (0.121)
Stimulants/energy boosters	61.4% (0.512)	72.5% (0.086)	44.4% (0.237)	48.1% (0.231)	0.0% (0.013) *	35.0% (0.026) *
Non-Vitamin/Mineral Antioxidants	53.4% (0.119)	65.2% (0.022) *	22.2% (0.018) *	44.4% (0.486)	40.0% (0.802)	25.0% (0.023) *
Fatty acids	58.0% (0.023)	79.7% (0.005) *	27.8% (<0.001) *	74.1% (0.487)	80.0% (0.767)	40.0% (0.023) *
Herbs and botanicals	68.2% (0.689)	76.8% (0.028) *	44.4% (0.036) *	81.5% (0.080)	40.0% (0.200)	40.0% (0.008) *
Fat burners/weight loss	69.3% (0.217)	75.2% (0.216)	33.3% (0.012) *	70.4% (0.761)	20.0% (0.099)	40.0% (0.044) *

* Numbers in the parenthesis represent Chi-Square test *p*-values. *p*-values < 0.05 show statistically significant difference.

**Table 5:** Logistic regression analysis of the statistically significant independent predictors of dietary supplement use.

Model	B	S.E. *	Significance *	Exp(B) *	95% CI for Exp(B) *	
Age vs. protein supplement use	1.334	0.607	0.028	3.796	1.156	12.469
Dietitian visit vs. vitamins use	−1.852	0.559	<0.001	0.157	0.052	0.469
Dietitian visit vs. carbohydrates use	−2.106	0.381	<0.001	0.122	0.058	0.257
Ethnicity vs. frequency of vitamin use	−0.625	0.278	0.024	0.535	−1.170	−0.081
Dietitian visit vs. frequency of carbohydrate use	−1.830	0.334	<0.001	0.160	−2.485	−1.175
Ethnicity vs. length of vitamin use	−0.788	0.278	0.005	0.455	−1.332	−0.244
Ethnicity vs. length of protein use	−0.681	0.275	0.013	0.506	−1.219	−0.143

* S.E: standard error, Significance: *p*-values < 0.05 show statistically significant difference, Exp (B): odds ratio, CI: confidence interval.

## Data Availability

Data is unavailable due to privacy or ethical restrictions.
